# Faux anévrysme de l´arcade palmaire chez un enfant: à propos d´un cas

**DOI:** 10.11604/pamj.2021.39.4.28933

**Published:** 2021-05-03

**Authors:** Fatima-Azzahra Samouh, Nadiha Igue Ossouka, Mohamed Labied, Dalal Laoudiyi, Kamilia Chbani, Siham Salam, Lahcen Ouzidane

**Affiliations:** 1 Pediatric Radiology Department, Ibn Rochd University Hospital, Faculty of Medicine and Pharmacy of Casablanca, Casablanca, Morocco

**Keywords:** Faux-anévrysme, arcade palmaire, échographie-doppler, à propos d´un cas, False aneurysm, palmar arch, doppler ultrasound, case report

## Abstract

Le pseudo anévrysme de l'arcade palmaire superficielle est une entité rare et seuls quelques cas ont été décrits dans la littérature. La majorité se développe après une lésion de l'artère suite à un traumatisme pénétrant, à une intervention chirurgicale antérieure ou à une ponction artérielle. Le diagnostic repose sur une suspicion clinique élevée, notamment en présence d'une masse pulsatile. Ci-dessous, nous présentons le cas d'un faux anévrisme de l'arcade palmaire superficielle chez un enfant de 3 ans à la suite d'une blessure par du verre brisé.

## Abstract (en)

Superficial pseudoaneurysms of the palmar arch is a rare disorder and only a few cases have been described in the literature. In the majority of cases it develops after artery lesion following penetrating injury, previous surgery or arterial puncture. Diagnosis is based on strong clinical suspicion, especially in patients with pulsatile mass. We here report a case of false aneurysm of the superficial palmar arch following broken glass injury in a 3-year old child.

## Introduction

Les pseudoanévrysmes des arcades palmaires sont très rares. Le mécanisme étiologique est souvent un traumatisme direct unique. Il est important d´identifier ces lésions, qui peuvent présenter des complications graves telles qu´une thrombose distale avec ischémie digitale ou une gangrène. Le diagnostic est généralement fait de façon aisée, devant une masse pulsatile, par une étude échographique couplée à un examen doppler.

## Patient et observation

**Figure 1:**
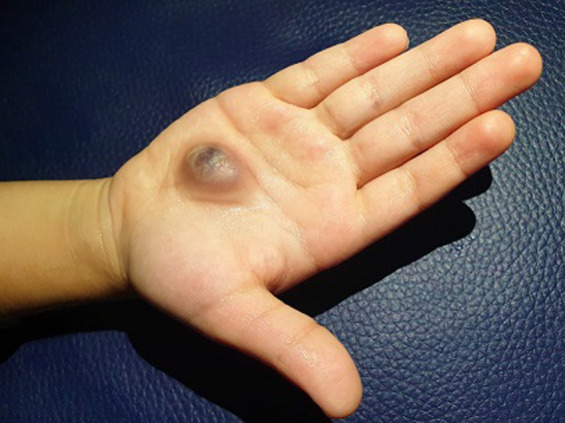
vue de face montrant une masse de l´hypothénar droit

Nous présentons le cas d´un enfant âgé de 3 ans, ayant comme antécédent un traumatisme pénétrant de la paume de la main droite, par un morceau de verre brisé, négligée par un soin à domicile. L´enfant a ensuite développé une masse enflée, pulsatile, douloureuse (Figure 1). Quatre semaines après la lacération, il a consulté un chirurgien orthopédique et a donc été référée à notre service pour examen échographique. Après consentement de la mère, l'examen physique a révélé une perfusion adéquate aux doigts sans atteinte fonctionnelle ou sensitif.

**Figure 2:**
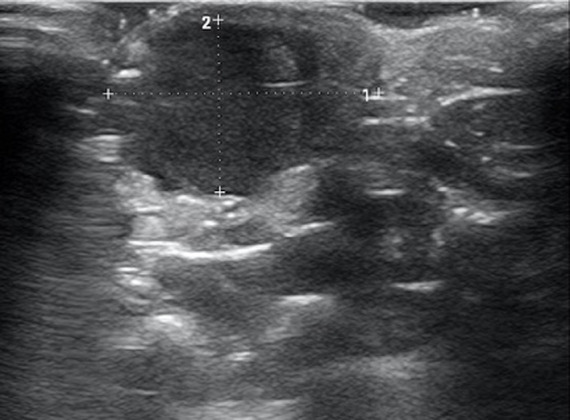
image échographique en mode B montrant une masse de 17x12x10mm, contenant un matériel thrombotique hémi-circonférentiel

**Figure 3:**
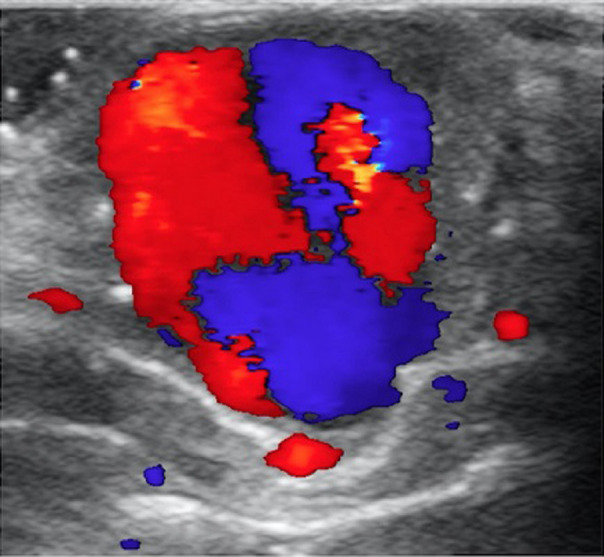
en mode doppler le caractère vasculaire circulant de la lumière est objectivé par le signe de Ying-Yang

Une échographie en mode duplex a été réalisée, révélant une masse pulsatile contenant un matériel thrombotique pariétal (Figure 2). Le signe de « Yin-Yang » en mode doppler couleur retrouvé, (Figure 3) est un élément déterminant de diagnostic en échographie. Un faux anévrysme de l'arcade palmaire superficielle a été diagnostiqué chez l´enfant.

La chirurgie a été réalisée sous anesthésie locale. La masse a été disséquée du tissu environnant et ligaturée à sa base. Aucune anticoagulation n'a été administrée pendant ou après la chirurgie. Aucun examen histologique n´a été indiqué car il était évident, de par la nature de la lésion, qu'il s'agissait d'un faux anévrisme. Le patient a été renvoyé à la maison le même jour. Après 9 mois de suivi basé exclusivement sur une évaluation clinique, le patient ne présentait plus aucune plainte, sa plaie était parfaitement guérie avec une fonction de la main intacte, des mouvements et des sensations normales et aucun engourdissement.

## Discussion

Les anévrismes des artères de la main sont des lésions rares. Il existe deux types: les anévrismes vrais suite à des microtraumatismes répétés ou à des artériopathies et les pseudo-anévrismes qui surviennent en post-traumatique ou iatrogène après cathétérisme [1]. Les faux et les vrais anévrysmes de l´arcade palmaire sont peu fréquents surtout chez les enfants. Ils sont le plus souvent d´origine traumatique, le diagnostic est alors facile, ou après des microtraumatismes répétés. Le diagnostic est évoqué en présence de signes de complications. Les observations rapportées dans la littérature sont peu nombreuses particulièrement chez l´enfant [2]. L´arcade palmaire peut être soumise à: un traumatisme direct, violent, responsable d´une rupture artérielle complète ou sous-adventitielle. Un faux anévrysme peut se développer, résultant de l´organisation d´un hématome au contact de cette solution de continuité. Il s´agit d´anévrysmes plutôt sacciformes [3]; de microtraumatismes répétés exposant au développement de lésions pariétales de type dysplasique, avec évolution anévrysmale (anévrysme fusiforme).

L´évolution de ces lésions anévrysmales est émaillée de complications à type de thrombose et d´emboles distaux, raison pour laquelle la présentation clinique est souvent d´allure vasculaire, mais aussi des complications nerveuses par compression de rameaux de la branche superficielle du nerf ulnaire. L´expression clinique dépend des circonstances. Une notion de traumatisme direct, associé à l´apparition d´une masse pulsatile de l´éminence hypothénar, suffit à évoquer le diagnostic, même en l´absence d´autres signes. Une masse pulsatile est présente dans 50% des cas [4]. Les signes cliniques sont d´autant plus nets que l´anévrysme est thrombosé et que le réseau anastomotique entre artère radiale et arcade palmaire superficielle est inexistant [5]. La qualité de ces anastomoses est évaluée par le test d´Allen qui garde toute sa valeur. Ce test a été réalisé par Stocker et Kosak chez un jeune adolescent de 16 ans, qui avait présenté une compression nerveuse dans le canal de Guyon secondaire à un faux anévrysme ulnaire post-traumatique de l´éminence hypothénar par un morceau de verre [6].

Le diagnostic d'un pseudoanévrysme peut être réalisé avec des méthodes invasives et non invasives: échodoppler couleur, angioscanner, angiographie par résonance magnétique et angiographie conventionnelle [3]. En utilisant l´imagerie en mode B, qui est un moyen simple, fiable, non invasif; un pseudoanévrysme apparaît généralement sous la forme d´une lésion anéchogène communiquant avec l´arcade palmaire; Ce mode est utile pour mesurer la taille du pseudoanévrysme et la compression de structures adjacentes [3]. L´examen échographique présente également d´autres avantages: il s´agit d´une méthode simple, rapide, peu coûteuse et utile pour évaluer les pseudoaneurysymes. L'échodoppler est le test diagnostique de choix et met en évidence un flux à haute vitesse via un défaut de la paroi artérielle lors de l'imagerie en flux de couleur. C´est cependant une technique qui dépend de l´opérateur.

Les diagnostics différentiels du pseudoanévrysme sont l´abcès, l´hématome et le kyste. Le seul point qui porte à discussion reste l´attitude thérapeutique. Certains auteurs prônent le traitement chirurgical systématique avec revascularisation. L´avantage est d´éviter d´exposer le patient aux complications emboliques [7]. La revascularisation par résection-anastomose termino-terminale est possible, le pontage veineux également. La thrombose du pontage reste possible, avec un résultat clinique fréquemment correct. La bonne tolérance est alors à rapprocher des cas d´anévrysmes thrombosés qui peuvent rester asymptomatiques [8]. L´abstention thérapeutique est possible, surtout si le patient est asymptomatique et l´anévrysme petit [7]. Par contre, en présence d´une masse douloureuse ou gênant l´activité professionnelle ou sportive, en présence de signes neurologiques, la résection de l´anévrysme est impérative. Seule la revascularisation est à discuter [4]. Une simple résection sans revascularisation est tout à fait envisageable, notamment en cas d´anévrysme thrombosé [9]. Pour certains, la revascularisation en cas de perméabilité de l´anévrysme est la meilleure attitude, pour d´autres, une ligature simple est possible [9].

## Conclusion

Le pseudo-anévrisme de l´arcade palmaire superficielle est une entité rare. Le diagnostic est échographique; le traitement fait classiquement appel à une réparation artérielle.
